# The Changes of Angiogenesis and Immune Cell Infiltration in the Intra- and Peri-Tumoral Melanoma Microenvironment

**DOI:** 10.3390/ijms16047876

**Published:** 2015-04-09

**Authors:** Vladimir Zidlik, Svetlana Brychtova, Magdalena Uvirova, Dusan Ziak, Jana Dvorackova

**Affiliations:** 1 CGB Laboratory, a.s., Laboratory of Molecular Genetics and Pathology, AGEL Research and Training Institute—Ostrava-Vitkovice Branch, Korenskeho 10, Ostrava 71000, Czech Republic; E-Mails: zidlik@pathology.cz (V.Z.); uvirova@pathology.cz (M.U.); ziak@pathology.cz (D.Z.); jana.dvorackova@fno.cz (J.D.); 2 Institute of Clinical and Molecular Pathology, Faculty of Medicine and Dentistry, Palacky University Olomouc, Hnevotinska 3, Olomouc 77515, Czech Republic; 3 Department of Pathology, Faculty of Medicine, University of Ostrava, Syllabova 19, Ostrava 70300, Czech Republic

**Keywords:** malignant melanoma, angiogenesis, nestin, microvascular density, CD90/Thy1, FOXP3

## Abstract

Malignant melanoma (MM) urgently needs identification of new markers with better predictive value than currently-used clinical and histological parameters. Cancer cells stimulate the formation of a specialized tumor microenvironment, which reciprocally affects uncontrolled proliferation and migration. However, this microenvironment is heterogeneous with different sub-compartments defined by their access to oxygen and nutrients. This study evaluated microvascular density (MVD), CD3^+^ lymphocytes (TILs) and FOXP3^+^ T-regulatory lymphocytes (Tregs) on formalin-fixed paraffin-embedded tissue sections using light microscopy. We analyzed 82 malignant melanomas, divided according to the AJCC TNM classification into four groups—pT1 (35), pT2 (17), pT3 (18) and pT4 (12)—and 25 benign pigmented nevi. All parameters were measured in both the central areas of tumors (C) and at their periphery (P). A marked increase in all parameters was found in melanomas compared to nevi (*p* = 0.0001). There was a positive correlation between MVD, TILs, FOXP3^+^ Tregs and the vertical growth phase. The results show that MVD, TILs and FOXP3^+^ Tregs substantially influence cutaneous melanoma microenvironment. We found significant topographic differences of the parameters between central areas of tumors and their boundaries.

## 1. Introduction

Cutaneous malignant melanoma (CMM) is highly aggressive with poor prognosis and high resistance to therapy. Further, prognosticators remain controversial and are generally based on the evaluation of the mitotic rate, regression, tumor-infiltrating lymphocytes (TILs) and growth phase [1]. Hence, there is an urgent need to identify new markers with more reliable predictive values than traditional clinical and histological parameters. Currently, potential reliable markers are a theme of intensive research. In malignant melanoma, like other solid cancers, tumor-stroma interactions that involve complex multiple cellular and molecular factors substantially affect their biological behavior [2,3]. Interactions between melanoma cells and other cell types in the microenvironment are mediated by endocrine and paracrine communication or through direct contact via cell-cell and cell-matrix adhesion, gap or tight junctional intercellular communication. Within a tumor, there are subcompartments with different microenvironmental milieus defined by their access to oxygen and nutrients. Therefore, different cancer cells within a tumor face different microenvironments [4].

A hallmark of solid tumor is abnormal vasculature, known as tumor angiogenesis, which is characterized by the new formation of vascular channels that enhance tumor cell proliferation, local invasion and distant metastasis. Tumor angiogenesis is uncontrolled and, in time, an unlimited process, involving the transition from the avascular to the vascular phases [5,6]. Tumor angiogenesis enhances the supply of oxygen and nutrients to solid tumor cells, which enables them to grow more rapidly and easily when vessels are formed in close proximity. It has been documented that new blood vessel formation is required after tumors attain a size of 1–2 mm [5]. Melanoma neovascularization has been correlated with poor prognosis, ulceration and an increased rate of relapse [6]. Recent studies showed that an effective marker for *in vivo* tumor angiogenesis is nestin, an intermediate filamentous protein that is considered to be a marker of endothelial proliferation [7]. Furthermore, it is also a marker of neuroectodermal stem and progenitor cells, because it is abundantly expressed in proliferating cells during embryonic development [8,9]. As a novel marker for activated blood, as well as lymphatic vessels, thymus cell antigen (CD90/Thy1) has been identified. CD90/Thy1 is a glycophosphatidylinositol-anchored, strongly-glycosylated protein that is expressed on the cell surface and belongs to the class of the immunoglobulin superfamily. It was originally identified as a thymocyte antigen and is a pan T-cell marker in mice. It is also known to be expressed by neurons and fibroblasts [10]. The molecule is expressed exclusively on endothelial cells (EC) at sites of inflammation or tumors, showing signs of activation. In contrast, there was no expression of Thy1 on the cell surface of resting EC in healthy tissues [11,12,13]. Today, it is thought to be an activation-associated cell adhesion molecule of human dermal microvascular endothelial cells to tumor cells. The mechanisms of tumor cell adhesion to the endothelium and the subsequent invasion into the surrounding tissue share similarities with the interaction occurring during leukocyte extravasation at sites of inflammation [11,13].

The morphological gold standard for assessing the neovasculature in human tumors has become microvascular density (MVD). This method requires the use of specific markers that highlight the vascular endothelium using immunohistochemical procedures. MVD in primary tumors is significantly associated with metastasis and poorer prognosis in several tumors and is the most predictive in those tumors that induce significant angiogenesis, namely carcinomas of breast and prostate and hematological malignancies [4].

An integral component of the tumor microenvironment is an inflammatory infiltrate, with a wide range of effects, which can act as a double-edged sword. On the one hand, immune cells have been reported to regulate malignant cells, and on the other hand, they may also have tumor-promoting effects. It has been reported that the infiltration of different human malignancies, e.g., ovarian, colorectal and breast with CD8^+^ T lymphocytes is associated with favorable prognosis [14]. Natural killers, dendritic cells and macrophages may also be considered as independent good prognostic indicators in different human cancers [14,15]. Conversely, malignant cells have been documented to create an immunosuppressive microenvironment. In this way, immune cells may help them escape immune surveillance and promote tumor progression. Increasing attention is currently paid to regulatory T-cells (Tregs), which are a subpopulation of CD25^+^CD4^+^ T lymphocytes with suppressive functionality [16]. The forehead transcriptional factor FOXP3 has been identified as a key regulator in the development and proper function of these cells, and it is also the only definitive marker [11,17]. In healthy individuals, the role of Treg is necessary in maintaining immunological tolerance and preventing autoimmune diseases. Activation of Tregs has been shown to lead to inhibition of cytotoxic CD8^+^ T lymphocytes and NK cells [17]. However, the role of Tregs in cancer development and progression is not clear. A large number of studies have shown that Tregs promote tumor growth by inducing host tolerance against tumor antigens by dampening the T-cell-mediated immune response against the tumor cells and enabling tumor cells to evade anti-tumor immunity. FOXP3 expression in cancers is thus associated with worse overall survival. Moreover, therapeutic inhibition of Tregs was shown to weaken their immunosuppressive effect and improve the course of the disease [14,18]. In malignant melanomas, FOXP3^+^ Treg is thought to be predictive of patient survival as a marker of early metastatic propagation [14].

The objectives of this study were to evaluate MVD with a focus on nestin, CD90-positive vessels and quantification of FOXP3^+^ Tregs in comparison to the numbers of CD3^+^ tumor infiltrating lymphocytes. To examine topographic differences, two distinct areas were analyzed in each lesion, the central area and the peripheral one, at the edge of the tumor adjacent to normal tissues.

## 2. Results and Discussion

### 2.1. Results

All obtained results with the Mann–Whitney *U*-test statistical analysis are summarized in Table 1.

**Table 1:** The table shows a comparison of the results between groups of melanomas (Stages pT1–pT4) and pigmented nevi in the central (C) and at the peripheral (P) areas. The results were statistically evaluated using the Mann–Whitney *U*-test (*p*-values).

Evaluated Parameters	Melanomas (*n* = 82)					Nevi (*n* = 25)					*p*
	Median	Q_1_	Q_3_	Min.	Max.	Median	Q_1_	Q_3_	Min.	Max.	
Nestin C (mm^2^)	10	6.75	20	0	62	4	2	9	0	26	0.0001
Nestin P (mm^2^)	22	13.75	38	2	78	4	1.5	8.5	0	17	<0.0001
FOXP3 C (mm^2^)	30	6.75	75.75	1	192	5	2	10.5	1	36	<0.0001
FOXP3 P (mm^2^)	9.5	2	18	1	160	1	1	1	1	21	<0.0001
CD3 C (mm^2^)	141	66	425	5	1330	38	21	55	2	158	<0.0001
CD3 P (mm^2^)	233.5	153	480	40	980	22	10	31	2	125	<0.0001
CD90 C (mm^2^)	0	0	3	0	15	0	0	0	0	4	<0.0001
CD90 P (mm^2^)	0	0	1	0	15	0	0	0	0	2	<0.0001
CD3 C/FOXP3 C	6.12	2.21	19.03	0.08	265	7.67	3.32	14.00	0.18	58	0.611
CD3 P/FOXP3 P	33.25	12.23	101.07	2.97	630	20.00	4.70	30.00	2.00	52	0.002

Abbreviations in the table: Q_1_ = the first quartile, Q_3_ = the third quartile, Min. = minimal value, Max. = maximal value.

#### 2.1.1. Microvascular Density with Anti-Nestin Antibody

**Figure 1:**
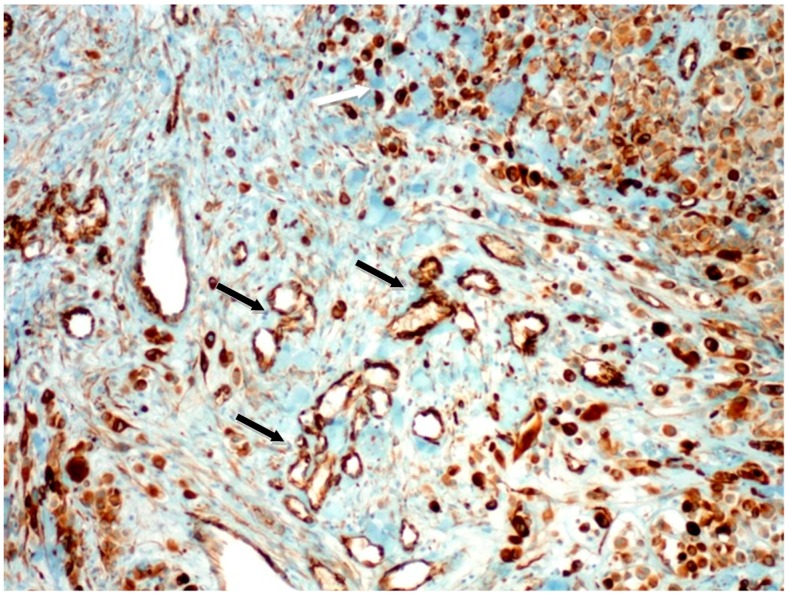
Strong nestin positivity in blood vessels (black arrows) at the edge of malignant melanoma (pT4 stage) (melanoma cells marked by white arrow); original magnification: 100×.

**Scheme 1:**
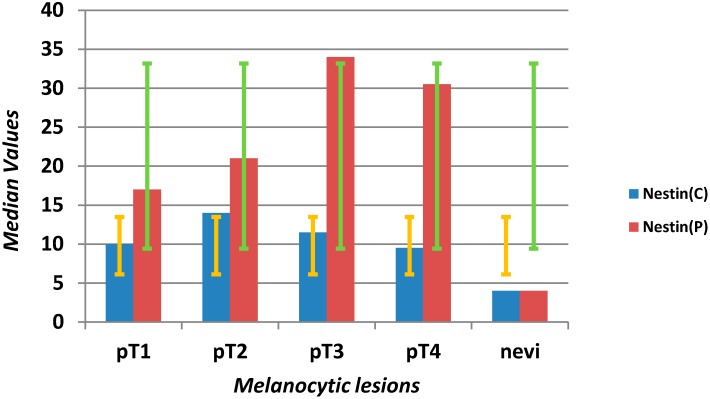
Evaluation of median values (*y*-axis) and error bars with the standard deviation of microvascular density (MVD) using anti-nestin antibody in the center (C) and at the periphery (P) in different stages of melanomas (pT1–4) and benign nevi (*x*-axis).

The microvascular density was quite low in benign nevi, ranging from 0 to 26 (median 4/mm^2^). A marked increase was observed in a group of melanomas, with MVD from 2 to 78, median 10 in the center and 22 at the edge, confirming a significantly higher density of nestin-positive vessels (*p* = 0.0001) both in the center and at the edge of tumors (Figure 1; Scheme 1).

Positive correlation (*p* = 0.0001) was found between MVD at the tumor periphery and the depth of invasion, with median values of 17, 21, 34, 31 for pT1, pT2, pT3 and pT4 groups, respectively. Central areas exhibited very similar MVD values in each group, with a median of 10–14 and no statistical significance.

#### 2.1.2. Microvascular Density with Anti-CD90 Antibody

**Figure 2:**
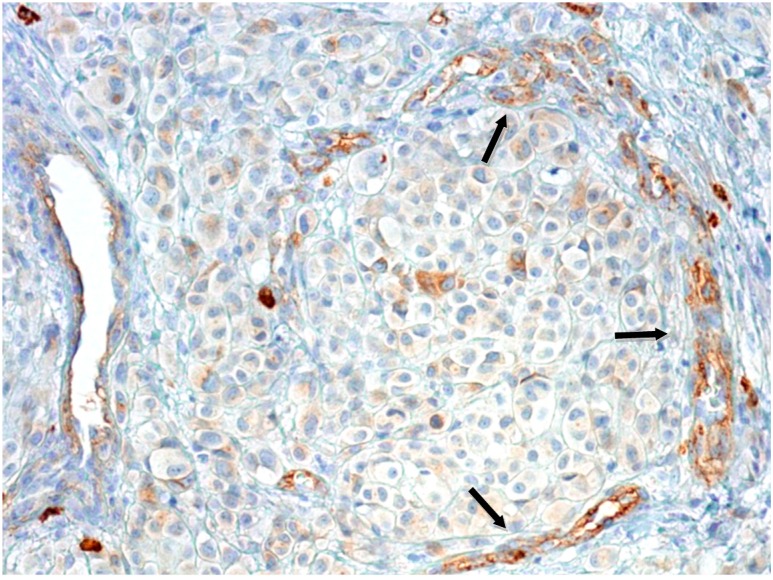
Moderate CD90 positivity of blood vessels (black arrows) in the central area of malignant melanoma (pT4 stage); original magnification: 100×.

**Scheme 2:**
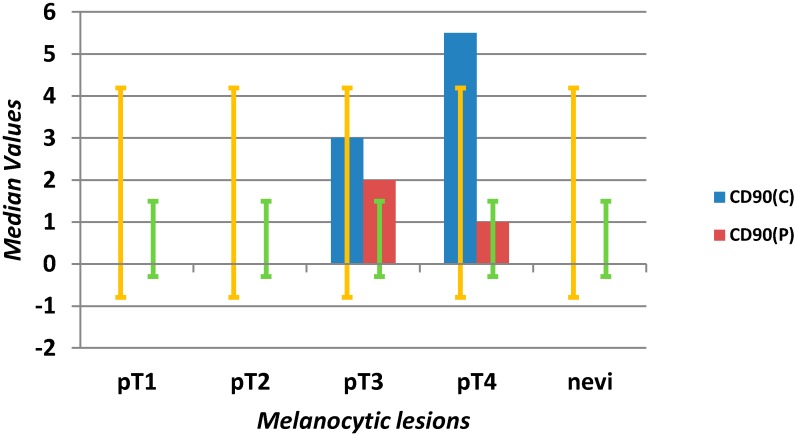
Evaluation of median values (*y*-axis) and error bars with the standard deviation of MVD using the anti-CD90 antibody in the center (C) and at the periphery (P) in different stages of melanomas (pT1–4) and benign nevi (*x*-axis).

No CD90 positive vessels were detected in nevi. In melanomas of the pT1 and pT2 stages, we found only individual vessels, both in the center and at the periphery (median zero). A significant increase (*p* = 0.0001) in CD90+ vasculature found for advanced tumors was predominantly intra-tumor vessels. Medians for pT3: three for the center, one for the periphery, pT4: 5.5 for the center and one for the periphery (Figure 2, Scheme 2).

#### 2.1.3. Tumor-Infiltrating Lymphocytes

**Scheme 3:**
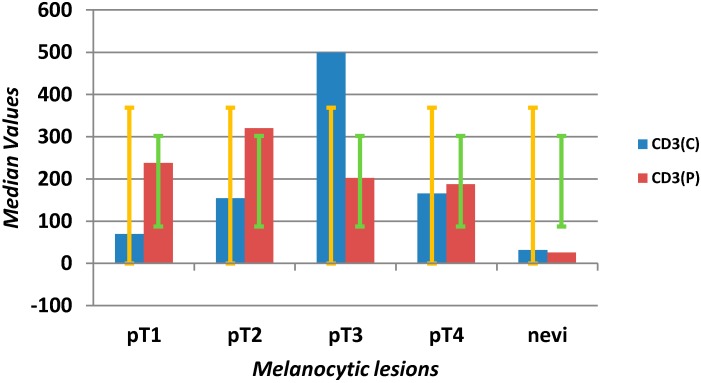
Evaluation of median values (*y*-axis) and error bars with the standard deviation of CD3^+^ T lymphocytes in the center (C) and at the periphery (P) of the microenvironment in different stages of melanomas (pT1–4) and benign nevi (*x*-axis).

The numbers of CD3^+^ T lymphocytes in nevi ranged from one to 158, median 38, inside the lesion and 22 at the edge. In melanomas, there was a significant increase from 2 to 1330 elements per 1 mm^2^ (*p* = 0.0001), with a median of 141 in central areas and 234 at the periphery. A significant increase in CD3^+^ tumor infiltrating lymphocytes was found in pT2, pT3 and pT4 *versus* pT1 melanomas (*p* = 0.0005) (Scheme 3). The peripheral area revealed even lymphocytic numbers, without any variations.

**Scheme 4:**
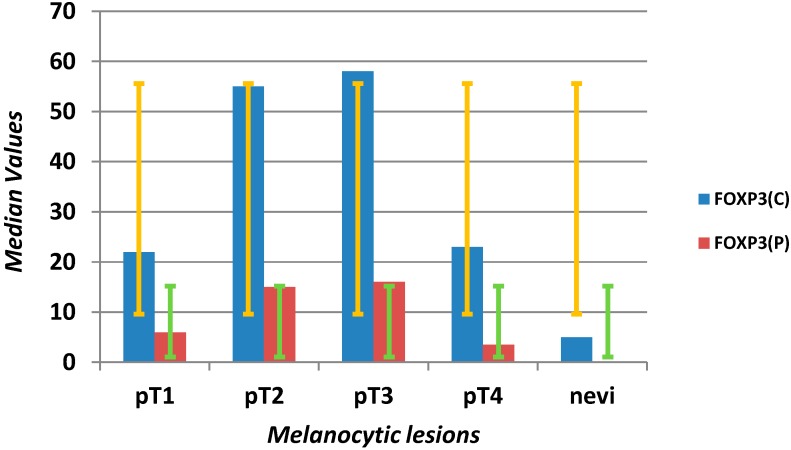
Evaluation of median values (*y*-axis) and error bars with the standard deviation of FOXP3^+^ T-regulatory lymphocytes in the center (C) and at the periphery (P) of the microenvironment in different stages of melanomas (pT1–4) and benign nevi (*x*-axis).

FOXP3^+^ Tregs were rare in pigmented nevi, with a median of five cells in the center, and one cell at the periphery. The numbers significantly increased in melanomas (*p* = 0.0001), from 1 to 192, median 30 in the center and 10 at the periphery (Scheme 4).

**Figure 3:**
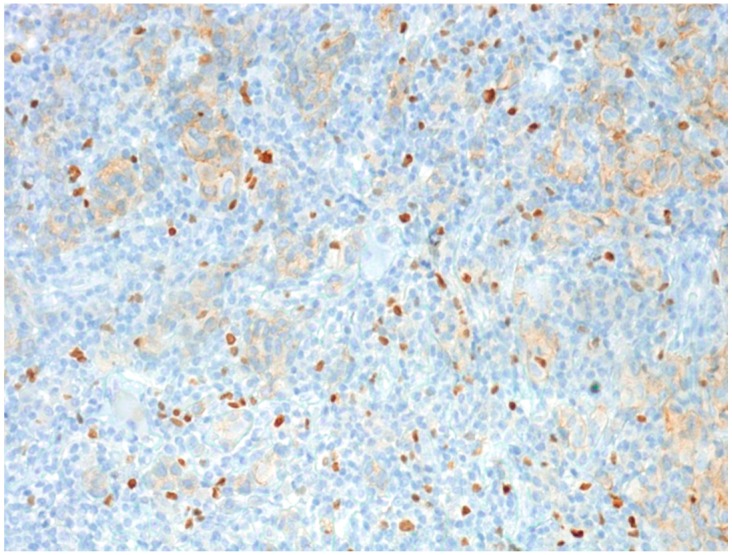
FOXP3-positive T-regulatory lymphocytes in the central region of malignant melanoma (pT3 stage); original magnification: 100×.

We also found differences in Tregs among individual melanoma groups, where the median Tregs for the pT1 group of melanomas was 22 in the center and six at the periphery, for pT2, 55 in the center, 15 at the periphery, for pT3, 58 in the center, 16 at the periphery and for pT4, 23 in the center and 3.5 at the periphery (Figure 3).

We found a significant higher number of Tregs in melanomas of pT2 *versus* pT1 (*p* = 0.015) and pT3 *versus* pT1 (*p* = 0.03). Surprisingly, in the pT4 group, a decrease in Tregs was observed in the center, as well as at the periphery.

**Scheme 5:**
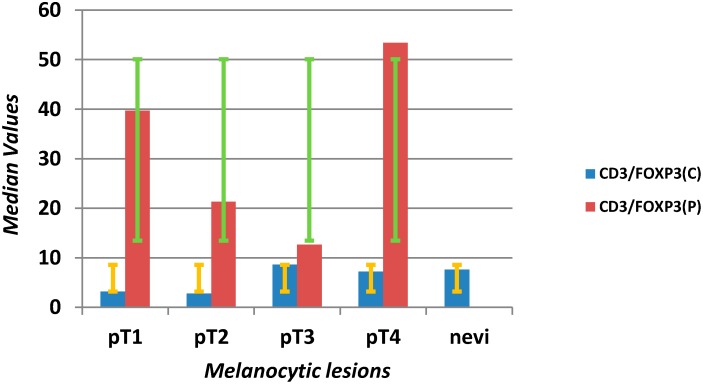
Evaluation of median values (*y*-axis) and error bars with the standard deviation of the CD3/FOXP3 ratio in the center (C) and at the periphery (P) of the microenvironment of different stages of melanomas (pT1–4) and benign nevi (*x*-axis).

The ratio of CD3/FOXP3^+^ Treg showed a significant shift for Tregs in pT2 and pT3 groups at the periphery of lesions (*p* = 0.005) (Scheme 5).

No associations were found with lymph node status or distant metastases.

### 2.2. Discussion

It has been determined that cancer progression is not solely determined by the characteristic of the tumor, but also by the host response [19]. CD8^+^ T-cells can be unquestionably heralded as one of the principal subsets of T-cells constitutively mediating an effective antitumor response. Activated T-cytotoxic lymphocytes can mediate specific destruction of tumor cells by the release of perforin and several types of granzymes, which are loaded in modified lysosomes [20,21]. CD4^+^ T lymphocytes are also an integral part of immunity, but their specific role in antitumor response remains unclear. They are known to facilitate cytotoxic T-cell (CTL) induction, although these cells have also been shown able to eliminate tumor cells in the absence of CD8^+^ T lymphocytes [22]. CD4^+^ T-cells have been documented to maintain a CTL response, too. During the last decade, a possible negative regulatory role of CD4^+^ T-cells has been described, and the existence of regulatory T-cells has been identified [14,17]. These cells represent about 6% of CD4^+^ T-cells and are present in peripheral blood and within the tumor environment. Antigen-specific activation and cell-cell contact were required for these clones of Tregs to exert suppressive activity. The presence of Tregs at tumor sites suggest that they could have a profound effect on the inhibition of T-cell effector responses against human cancers [17]. Besides anti-inflammatory cytokines, Tregs inside the tumor may repress immunity via other mechanisms. For example, they may inhibit T-cell proliferation. Whether the regulatory cells naturally exist in the host or whether they initially arrive as helper T-cells and only convert later is not altogether clear. Anti-tumor lymphocytes migrating to the tumor site may become compromised or may adversely adapt to the suppressive environment to promote growth instead of regression [16]. In agreement with these data, recent studies have revealed that the type, not the quantity of tumor-infiltrating cells seems to be a more critical determinant of prognosis. Since cancer is a disease caused by an array of various types of mutations, differences in T-cell subsets are not altogether surprising. Melanoma is one of those tumors known to possess the ability to elicit a profound immune response. Some data show that the induction of a strong immune response in patients with melanoma may improve survival [18,23]. Numerous immune-based therapies (involving cytokines, antibodies, cancer vaccines, adoptive immunotherapy and combinations of these therapeutic agents and modalities) are the focus of studies on alternative therapeutic approaches. Although cancer vaccines and adoptive T-cell transfer have been shown to increase the levels of the circulating tumor antigen-specific T-cells, these approaches produce clinical responses in only a few patients [17]. Recent studies have suggested that the presence of FOXP3^+^ Tregs in the tumor microenvironment, the expression of inhibitor ligands on melanoma cells, the secretion of immunosuppressive factors by melanoma cells and the activity of nutrient-catabolizing enzymes may contribute to the resistance of the tumor to immune destruction. It has been reported that high numbers of circulating Tregs are associated with rapid tumor progression in experimental animal models of melanoma and in patients with melanoma. In these patients, the presence of FOXP3^+^ cells in primary tumor has also been associated with a higher frequency of metastases in the sentinel lymph node [12,24]. On the other hand, the blocking of normal mechanisms responsible for the downregulation of immune responses has been shown to improve melanoma outcome efficiently [14]. In our study, we focused on evaluating the FOXP3^+^ Tregs, as well as the CD3^+^/Treg ratio. While the density of these cells was very low in benign nevi, we confirmed their increase in melanomas, both inside and at the tumor edge. It was postulated that a major determinant of immune cell infiltration may be the stage of disease, where host immune response may decrease with increasing tumor growth [25]. In agreement with this finding is increased FOXP3^+^ Tregs in pT2 and pT3 melanoma stages in our study, with the most pronounced changes in the CD3^+^/Treg ratio at the periphery of tumors. The increase in Tregs density may represent a mechanism of tumor resistance to immune destruction, creating an immunosuppressive melanoma microenvironment. Surprisingly, pT4 melanomas exhibited lower Tregs values and high a CD3^+^/Treg ratio, particularly at the periphery. We suggest that low numbers of FOXP3^+^ accompanied by high TIL numbers may paradoxically be a feature of tumor progression, as was described for colorectal carcinomas [26]. The presence of cytotoxic T lymphocytes in advanced tumors may be a consequence of the greater production of abnormal peptides resulting in altered DNA repair, a typical feature of the genetic instability of malignancies [26,27]. Moreover, genetically-unstable tumors are often HLA class I-negative and might escape T-cell-mediated immune killing [19].

It has been well documented that angiogenesis is crucial for cutaneous melanoma progression, where melanoma neovascularization has been correlated with poor prognosis and an increased rate of relapse [6]. A possible explanation is that the increased vasculature enhances the chance for tumor cells to enter the circulation. Moreover, newly-formed vessels or capillaries have leaky and weak basement membranes, through which tumor cells can penetrate more easily than mature vessels [28]. Angiogenesis is a complicated and dynamic process, whose measurement in tissue provides only a snapshot, not straightforward views of tumors. Despite its limitation, microvascular density (MVD) counting has become the morphological standard for assessing the neovasculature in human tumors, with prognostic and predictive impact [4]. MVD seems to correlate with outcome, especially in high-grade tumors. It is widely assumed that tumors with high MVD are good candidates for clinical trials of antiangiogenic therapies, whereas tumors with typically low MVD are thought to be poor candidates for such clinical trials. Nevertheless, despite the initial confirmatory publications, numerous reports fail to show a positive association between increasing tumor vascularity and reduced tumor outcome [4,29]. One has to consider that heterogeneous methodologies used to calculate MVD among different studies might play a role. However, other factors have to be considered, too, such as tumor topography and functional changes in the endothelium. Topography is important in the differentiation of tumor vessels into those supplying the invading tumor edge and those serving the inner tumor area.

As adhesive interactions between tumor cells and endothelium are critical steps in tumor metastasis, it is not surprising that functionally- and phenotypically-changed endothelium may substantially contribute to cancer progression. In accordance with previous explorations, we confirmed significantly higher MVD counts in melanomas *versus* benign tissue [30]. Moreover, we found markedly-enhanced vascularization in advanced pT3 and pT4 melanomas. As far as the predictive role of MVD is concerned, we cannot confirm and direct association, as none of our tumors within a five-year follow-up formed either distant metastasis or relapsed. However, a lack of correlation between MVD and tumor outcome was described in sinonasal, oral and canine melanomas, too [14,31]. In our study, we focused on activated, proliferating endothelium, using antibodies to highlight it—nestin and CD90/Thy1—instead of the widely-used CD31 or CD34 [28,32].

In this study, we found higher MVD of nestin-positive vessels in melanomas *versus* nevi, especially in advanced tumors. Although areas of hot spots were not infrequently seen within the inner tumor area, they usually predominated at the tumor edge—the zone of tumor/normal tissue interaction. Peripheral tumor areas are composed of typical capillaries derived from pre-existing vessels. Central areas of tumors, on the other hand, are at least partly made up of tube-like endothelial structures, known as vasculogenic mimicry (VM), that are generated directly by the tumor cells [15]. The molecular mechanisms that underlie VM are not fully clear, but metalloproteinases via their cleavage of laminin, E-cadherin by promoting adherence of the VM channel wall to tumor cells, tumor cell dedifferentiation and tumor microenvironment have been shown to play a role in VM. A three-stage phenomenon among VM channels, mosaic blood vessels and endothelium-dependent blood vessels has been proposed, where all three patterns participate in tumor blood supply. These facts may explain why therapeutic strategies targeting endothelial cells have no effect on tumor cells [6]. They may also partly explain why MVD measurement is not a direct predictor of anti-angiogenic therapy [4].

A good candidate for the detection of functionally-altered vessels seems to be CD90/Thy1. This molecule plays an important role in the adhesion of tumor cells to the endothelium and is associated with the specific interaction of the αvβ3 integrin on melanoma cells. This interaction mediates the binding melanoma cells to the endothelium. Blocking αvβ3 reduced the adhesion of αvβ3-expressing melanoma cells to the level of melanoma cells lacking αvβ3 [13]. Except for blood vessels, CD90/Thy-1 was found to be highly expressed on lymphatic endothelial cells [11,12]. We found no CD90 expression on endothelium of normal skins and nevi. Similarly, early-stage melanomas pT1 and pT2 had only very low numbers of CD90^+^ vessels. Advanced melanomas in pT3 and pT4 groups showed a significantly higher density of CD90-positive vessels, especially in central regions. These findings confirm phenotypically- and functionally-altered vascularization, especially in advanced-stage melanomas, and suggest a potential negative prognostic role of the protein in the disease.

## 3. Experimental Section

Archival cases of 82 cutaneous malignant melanomas and 25 benign pigmented compound or intradermal nevi were evaluated. Adult patients of both sexes, aged from 42 to 69, were included. The melanomas were divided according to the AJCC TNM classification for melanoma staging into four groups—pT1 (*n* = 35 melanomas), pT2 (*n* = 17 melanomas), pT3 (*n* = 18 melanomas) and pT4 (*n* = 12 melanomas) [33]. The corresponding H&E slides were first reviewed by the pathologist for confirmation of diagnosis and adequacy of the material. All selected tissue samples were formalin-fixed and paraffin-embedded. The study was performed on 5 µm-thick tissue sections by an indirect immunohistochemical method and stained in an automated immunostainer (VENTANA BENCHMARK XT, Ventana Medical System, Tucson, AZ, USA), in which all steps of the procedure were done. After deparaffinization, rehydration and blocking of endogenous peroxidase activity, all sections were incubated with a primary antibody at a room temperature. We used monoclonal mouse anti-nestin antibody (Millipore, Darmstadt, Germany, clone 10C2, Cat. #MAB5326, dilution 1:75, incubation time 20 min), monoclonal rabbit anti-FOXP3 antibody (Novus Biologicals, Cambridge, UK, clone SP97, NBP2-12498, dilution 1:150, incubation time 20 min), anti-CD3 (DakoCytomation, Glostrup, Denmark, polyclonal Rabbit Anti-human, Code 1580, dilution 1:50, incubation time 32 min) and rabbit monoclonal anti-CD90 antibody (RabMAbs, Abcam, Cambridge, UK, clone EPR3133, ab133350, dilution 1:100, incubation time 28 min). No primary antibody needed an antigen retrieval step. For detection, we used the VENTANA detection kit (VENTANA iVIEW™ DAB Detection Kit, Ventana Medical System, Tucson, AZ, USA, Catalogue No. 760-091), which is standardized to detect mouse IgG, IgM and rabbit IgG antibodies, without any further requirements on dilution or titration of the solutions. As a part of the kit, there is streptavidin-horseradish peroxidase complex conjugated to the biotin-bound secondary antibody, as well as hydrogen peroxide substrate and DAB (diaminobenzidine) for visualization. The whole set of cases was used for each analyzed marker. All parameters were evaluated by light microscopy counting capillary lumens, FOXP3^+^ and CD3^+^ lymphocytes per unit area of 1 mm^2^ in a “hot spot”—a field with the highest capillary density or the highest lymphocytic infiltrate. We counted at least two fields for each tumor. Both the central areas of tumors (C) and their periphery (P) were measured. The differences between malignant and benign melanocytic lesions were evaluated. In a group of melanomas, obtained data were compared with the depth of invasion, lymph node and distant metastases status.

The results were statistically evaluated using the Mann–Whitney *U*-test and Kruskal–Wallis test with Bonferroni correction. *p*-values of 0.05 or less were considered to be statistically significant.

## 4. Conclusions

In summary, the results show that MVD, TILs and FOXP3^+^ Tregs are substantially involved in the alteration of the cutaneous melanoma microenvironment. More marked changes were observed, especially in advanced stages of the disease. We also confirmed that there are significant topographic differences of the parameters between central areas of tumors and their boundaries. However, for determination of the analyzed parameters as unequivocal prognostic and predictive factors of melanoma, further studies are needed.
